# Comparison of Diagnostic Tests in Distinct Well-Defined Conditions Related to Dry Eye Disease

**DOI:** 10.1371/journal.pone.0097921

**Published:** 2014-05-21

**Authors:** Monica Alves, Peter Sol Reinach, Jayter Silva Paula, Antonio Augusto Vellasco e Cruz, Leticia Bachette, Jacqueline Faustino, Francisco Penteado Aranha, Afonso Vigorito, Carmino Antonio de Souza, Eduardo Melani Rocha

**Affiliations:** 1 Faculty of Medicine Ribeirão Preto, São Paulo University, Ribeirão Preto, SP, Brazil; 2 Pontific Catholic University of Campinas, Campinas, SP, Brazil; 3 Faculty of Medical Sciences, University of Campinas, Campinas, SP, Brazil; Medical University Graz, Austria

## Abstract

**Purpose:**

This study compares signs, symptoms and predictive tools used to diagnose dry eye disease (DED) and ocular surface disorders in six systemic well-defined and non-overlapping diseases. It is well known that these tests are problematic because of a lack of agreement between them in identifying these conditions. Accordingly, we provide here a comparative clinical profile analysis of these different diseases.

**Methods:**

A spontaneous and continuous sample of patients with Sjögren's syndrome (SS) (n = 27), graft-versus-host-disease (GVHD) (n = 28), Graves orbitopathy (n = 28), facial palsy (n = 8), diabetes mellitus without proliferative retinopathy (n = 14) and glaucoma who chronically received topical drugs preserved with benzalkonium chloride (n = 20) were enrolled. Evaluation consisted of a comprehensive protocol encompassing: (1) structured questionnaire - Ocular Surface Disease Index (OSDI); (2) tear osmolarity (TearLab Osmolarity System - Ocusense); (3) tear film break-up time (TBUT); (4) fluorescein and lissamine green staining; (5) Schirmer test and (6) severity grading.

**Results:**

One hundred and twenty five patients (aged 48.8 years-old±14.1, male:female ratio = 0.4) were enrolled in the study, along with 24 age and gender matched controls. Higher scores on DED tests were obtained in Sjögren Syndrome (P<0.05), except for tear film osmolarity that was higher in diabetics (P<0.001) and fluorescein staining, that was higher in facial palsy (P<0.001). TFBUT and OSDI correlated better with other tests. The best combination of diagnostic tests for DED was OSDI, TBUT and Schirmer test (sensitivity 100%, specificity 95% and accuracy 99.3%).

**Conclusions:**

DED diagnostic test results present a broad range of variability among different conditions. Vital stainings and TBUT correlated best with one another whereas the best test combination to detect DED was: OSDI/TBUT/Schirmer.

## Introduction

Dry eye disease (DED) is a multifactorial condition involving changes in tear composition and volume as well as ocular surface (OS) integrity, with a several different risk factors and symptoms. [5] It is well known that diagnostic test results for DED poorly correlate with one another and with symptoms. [6], [7] A possible reason for this disparity is that the heterogeneity in causative DED factors induces different changes in underlying mechanisms controlling lacrimal gland (LG) and OS physiology.

In Sjögren's syndrome (SS), the LG turns into a target of the immune system. Consequently, the presence of focal lymphocytic infiltrates leads to increased production of pro-inflammatory cytokines, acinar damage and aqueous production impairment. [8], [9] Similar inflammatory responses may be noted in graft versus host disease (GVHD) that is also accompanied by conjunctival inflammation, meibomian gland dysfunction and severe DED features.[10]

As LG secretion is under neural control, proper stimuli from the ocular surface afferent sensory nerves in the cornea and conjunctiva activate efferent responses to stimulate LG secretion. In this regard, conditions such as diabetes mellitus (DM) and facial palsy (FP) can be important causes of dysfunctional changes in tear volume and/or composition.[11]–[14] In addition, it is also important to consider that hormones, in particular, insulin, thyroid and sex steroid hormones are regulators of LG functions [15]–[18] and that the OS is constantly affected by their related diseases (*e.g*.; Graves orbitopathy). Finally, environmental factors, aging and topical medications or their preservatives (*e.g*., benzalkonium chloride or BAK), may also contribute to either improve or aggravate DED. [19]–[21]

Our hypothesis is that differences in the underlying mechanisms of diseases (*i.e*., SS, GVHD, DM, FP, Graves orbitopathy, BAK toxicity) affect in distinct ways and intensity tear secretion and DED clinical presentation. The present work compares the signs and symptoms of DED in six systemic well-defined and non-overlapping diseases. By evaluating the performance of DED tests, we draw attention to the need to deal with this challenging problem of diagnosing this highly prevalent and sight threating condition.

## Methods

A total of one hundred and twenty-five DED subjects were included in this study. Consecutive patients attending the outpatient DED clinic in a tertiary care university hospital were invited to participate. Patients presenting one of the following conditions associated with DED were included: SS (diagnosed following the American-European Criteria)[22], GVHD, Graves orbitopathy, facial palsy (based on clinical criteria), chronic glaucoma topical treatment with benzalkonium chloride (BAK) preserved drugs for at least one year, and diabetes mellitus without retinopathy (based on fasting glycemic levels and indirect fundoscopic evaluation).

All patients were under clinical treatment at the time of their evaluation. Due to a lack of agreement among the established DED diagnostic criteria, described in different clinical studies [1]–[4], we adopted the following criteria: Ocular Surface Disease Index (OSDI) score > 20 and/or Schirmer test (ST) <10 mm or tear break up time (TFBUT) ≤6 seconds and/or any of the vital staining >3 and/or tear film osmolarity >310 mOsm. DED diagnosis was considered if the patient presented at least one positive test according to these pre-established criteria. Patients were separated into six different subgroups based on their disease (i.e.; SS, GVHD, Graves orbitopathy, facial palsy, diabetes mellitus without retinopathy, or chronic glaucoma treatment with BAK preserved eye drops), were compared throughout the study.

Twenty-four healthy volunteers, matched by age and gender were enrolled as a control group. As exclusion criteria we considered: active ocular infection, ocular allergy, history of refractive surgery or contact lens wear, pregnancy and lactation, or conditions with clinical overlapping of the aforementioned diseases.

The study was approved by the Faculty of Medicine Ethics Committee, University of São Paulo and was conducted in accordance with the tenents of the Declaration of Helsinki and current legislation on clinical research. Written informed consent was obtained from all subjects after explanation of the procedures and study requirements. Evaluation of DED consisted of a protocol encompassing: OSDI questionnaire, tear film osmolarity measurement, tear break-up time (TFBUT), corneal staining with fluorescein, Schirmer test (ST) and conjunctival staining with lissamine green, as described below and according to the following sequence[6].

### 1. OSDI

The OSDI score is a subjective symptom questionnaire, used as DED outcomes measurement to estimate its severity[6], [23]. A portuguese language validated version was used. [24]

### 2. Tear film osmolarity

Tear film osmolarity was measured using a lab-on-a-chip system to simultaneously collect and analyze the electrical impedance of a tear sample (TearLab Corp San Diego, CA, USA). A small tear sample of (50-nanolitre) was collected from the lower meniscus, using a disposable test chip by passive capillary action. Osmolarity readings are given in milliosmoles per liter a few seconds after the transfer. [25] Quality control procedures were applied before starting patient testing each day, to confirm function and calibration according to the manufacturer instructions.

Slit lamp examinations inspected the cornea and conjunctiva at a magnification of 10–16X and were used to perform some of following tests as previously described. [6], [26]

### 3. Tear film break-up time

TFBUT was measured 10–30 seconds after instillation of 5 µl of a 2% sodium fluorescein solution (Allergan, Guarulhos, Brazil) and calculating the average of three consecutive breakup times, determined manually using a stopwatch (in seconds).

### 4. Corneal fluorescein staining

Corneal fluorescein staining was evaluated using cobalt blue illumination following the 15-point NEI/ Industry scale (grades of 0–3 for five regions of the ocular surface), after TFBUT measurments.

### 5. Schirmer test

Tear production was measured in both eyes simultaneously with Schirmer test strip for 5-minutes without anesthetic (Ophthalmos Ltd., São Paulo, Brazil).

### 6. Lissamine green conjuntival staining

Lissamine green conjuntival staining was evaluated after instilling 10 µl of a 1% sodium lissamine green dye (Ophthalmos Ltd., São Paulo, Brazil). Conjunctival staining assessment used a grading scheme described by van Bijsterveld according to a modified NEI/Industryscale, where grades of 0–3 are assigned for three regions (temporal, central and nasal).

All measurements were performed by the same investigators, under similar testing conditions and at room temperature. For each sign, the more severe measurement in the two eyes was used in the analysis of disease severity.

In this study, patients were using recommended treatments for their diseases and artificial tears for DED. Patients were instructed to not use any eye drops on the day when they were examined in the clinic.

Dry eye severity was graded according to a modified severity score scheme from 1–4 as described previously. [5].

### Statistics

Descriptive statistics for continuous data were reported as mean±SD. Continuous variables were compared using Kruskall-Wallis (with Dunn's post hoc test) when two or more than two groups were analyzed, respectively. Correlations between the variables under investigation were determined using Spearman correlation coefficient. Differences were considered significant at P<0.05. All analysis were performed using SPSS v.17.0 (IBM Corp., Armonk, NY, USA). The values of sensitivity, specificity, positive predictive value and accuracy were made for the following tests: OSDI>20, tear film osmolarity >310 mOsm, Schirmer test <10 mm, TFBUT<6 sec, and vital staining ≥3, as standardized above to include as DED for each condition. All calculations of true positive, true negative, false positive and false positive were made taking into consideration, except the one that is under observation. For the best combination of tests to detect DED in this population, we applied binary multivariate logistic regression through a backward model, including all individuals and test results.

We evaluated some statistical measures in order to better understand the performance of DED diagnostic tests. Sensitivity relates to a test's ability to identify positive results (i.e, cases of DED), is calculated with true positive results and total of true conditions; while specificity evaluates the ability to identify negative results (i.e., non DED) and is calculated using true negative results and total of negative conditions. Accuracy is used to correctly identify or exclude a condition, here DED. That is, the accuracy is the proportion of true results (both true positives and true negatives cases of DED) in the population. Positive Predictive Value (PPV) is also an indicator of accuracy, reinforcing the capacity of the test to identify the real positive result whereas Negative Predictive Value (NPV) identifies the real negative ones. For all those calculations, the study assumed that the cut-off values as standardized above to include DED for each condition. All calculations of true positive, true negative, false positive and false positive were made taking into consideration the exams, except the one that is under evaluation.

## Results

A total of one hundred twenty-five DED subjects, with a male:female ratio of 0.4, and mean age of 48.8±14.1 years were included in this study. The control group consisted of twenty-four normal volunteers, with a male:female ratio of 0.38, and mean age of 45.7±12.7 years. Based on the presence of baseline conditions associated with DED, the following subgroups were formed: SS (n = 27), GVHD (n = 28), Graves orbitopathy (n = 28), facial palsy (n = 8), diabetes mellitus without retinopathy (n = 14), and patients under chronic glaucoma treatment with BAK preserved eyedrops (n = 20).

Results obtained in the clinical and laboratory evaluation of patients and controls varied significantly among patients, controls and across different subgroups (Table 1).

**Table 1:** Descriptive statistics for clinical and laboratory parameters of DED in the study groups. Data expressed as mean±standard deviation (mimimum-maximum).

Group	Frequency of DED	OSDI (0–100)	Tear Film Osmolarity (mOsmos/L)	TFBUT (seg)	Schirmer Test (mm)	Fluorescein staining (0–15)	Lissamine staining (0–9)
**Control** (n = 24)	20% (19/24)	12.83±10.35 (0–45)	295.3±7.8 (284–308)	10.50±2.6 (5–10)	24.42±9.42 (12–40)	0.08±0.28 (0–1)	0.08 ±0.28 (0–1)
**SS** (n = 27)	100% (27/27)	57.27±21.66* (15–87)	316.1± 23.1* (275–382)	2.61±2.26* (0–10)	6.46±9.64* (0–35)	5.53±4.94* (0–15)	3.92±3.10 * (0–8)
**GVHD** (n = 28)	100% (28/28)	48.21±21.69 * (7–85)	310.7±18.26* (275–374)	3.96±2.84* (0–11)	8.39±8.68* (0–35)	4.35±3.78* (0–15)	2.32±2.42* (0–8)
**Graves orbitopathy** (n = 28)	100% (28/28)	47.14±18.83* (12–79)	300.0±15.81 (279–351)	5.39±3.15* (2–12)	15.71±12.33 (2–40)	0.67±1.21 (0–4)	1.14±1.26* (0–5)
**Glaucoma treatment with BAK** (n = 20)	90% (18/20)	33.60±21.42 (2–87)	301.2±25.39 (202–238)	5.38±3.38* (1–12)	15.58±13.98 (2–40)	0.60±1.14 (0–4)	0.60±0.82 (0–3)
**Facial palsy** (n = 8)	100% (8/8)	49.83±28.0* (13–97)	292.9±7.98 (285–308)	6.50±7.89 (0–20)	9.0±9.28* (0–25)	7.57±4.42* (2–15)	1.0±1.67 (0–4)
**Diabetes Mellitus without retinopathy** (n = 14)	100% (14/14)	29.29±19.55 (10–73)	318.6±15.86* (294–363)	6.14±4.13* (0–12)	11.82±2.86 (3–36)	1.14±2.03* (0–7)	0.78±1.52 (0–5)

*P<0.05.

Kruskall Wallis test.

Correlation coefficients calculated from the data set of all patients included in this study are reported in the Table 2. Among the diagnostic tests evaluated herein the coefficients were consistently modest suggesting lack of concordance. The highest values of correlation were observed between fluorescein and lissamine, and between TBUT and lissamine (R^2^ = 0.43 and R^2^ = 0.31, respectively). Similarly, the correlations coefficients evaluated within each study subgroup had a wide range of values for each test and no consistent relationship (Table 3).

**Table 2:** Correlation coefficients among DED tests grouping all subjects, from the 6 subgroups and controls.

	OSDI	Fluorescein	Lissamine	TBUT	Schirmer	Tear osmolarity
**OSDI**		R^2^ = 0.14* CI95% 0.23–052 p<0.0001	R^2^ = 0.17* CI95% 0.27–0.55 p <0.0001	R^2^ = 0.17* CI95%–0.55–0.27 p<0.0001	R^2^ = 0.09* CI95%–0.45–0.14 p = 0.0003	R^2^ = 0.07* CI95% 0.12–0.43 p = 0.0006
**Fluorescein**	R^2^ = 0.14* CI95% 0.23–052 p<0.0001		R^2^ = 0.43* CI95% 0.51–0.7 p<0.0001	R^2^ = 0.22* CI95%–0.59–0.33 p<0.0001	R^2^ = 0.17* CI95%–0.55–0.26 p<0.0001	R^2^ = 0.07* CI95% 0.11–0.42 p = 0.0007
**Lissamine**	R^2^ = 0.17* IC95%0.27–0.54 p<0.0001	R^2^ = 0.43* CI95%0.51–0.76 p<0.0001		R^2^ = 0.31* CI95%–0.66–0.43 p<0.0001	R^2^ = 0.17* CI95%–0.55–0.27 p<0.001	R^2^ = 0.13* CI95%0.21–0.50 p<0.0001
**TBUT**	R^2^ = 0.17* CI95%–0.55–0.27 p<0.0001	R^2^ = 0.22* CI95%–0.59–0.33 p<0.0001	R^2^ = 0.31* CI95% –0.66–0.43 p<0.0001		R^2^ = 0.24* CI95%0.36–0.62 p<0.0001	R^2^ = 0.09* CI95%–0.45–0.14 p<0.0001
**Schirmer**	R^2^ = 0.09* CI95% –0.45–0.14 p = 0.0003	R^2^ = 0.17* CI95%–0.55–0.26 p<0.0001	R^2^ = 0.17* CI95%–0.55–0.27 p<0.001	R^2^ = 0.24* CI95%0.36–0.62 p<0.0001		R^2^ = 0.04* CI95%–0.36–0.03 p = 0.01
**Tear osmolarity**	R^2^ = 0.07* CI95% 0.12–0.43 p = 0.0006	R^2^ = 0.07* CI95% 0.11–0.42 p = 0.0007	R^2^ = 0.13* CI95%0.21–0.50 p<0.0001	R^2^ = 0.09* CI95%–0.45–0.14 p<0.0001	R^2^ = 0.04* CI95%–0.36–0.03 p = 0.01	

*p<0.05. (n = 149 individuals). Spearman's correlation coefficient.

**Table 3:** Correlation coefficients of exams for DED in six, non-overlapping subgroups of patients.

	Sjogren	GVHD	Graves	Glaucoma	Facial palsy	Diabetes
**OSDI/Fluorescein**	R^2^ = 0.47* CI95%0.51–0.82 p<0.0001	R^2^ = 0.32* CI95% 0.35–0,73 p<0.0001	R^2^ = 0.07* CI95% 0.01–0.52 p = 0.04	R^2^ = 0.19* CI95%0.16–0.66 p = 0.002	R^2^ = 0.32* CI95%0.25–0.77 p = 0.001	R^2^ = 0.04 CI95% 0.12–0.5 p = 0.19
**OSDI/Lissamine**	R^2^ = 0.34* CI 95%0.36–0.75 p<0.0001	R^2^ 0.24* CI95% 0.25–0.68 p = 0.0002	R^2^ = 0.23* CI95%0.23–0.67 p = 0.0003	R^2^ = 0.28* CI95%0.26–0.71 p = 0.0002	R^2^ = 0.10 CI95%–0.06–0.6 p = 0.08	R^2^ = 0.017 CI95%–0.20–0.44 p = 0.4
**OSDI/TBUT**	R^2^ = 0.56* CI95%–0.86–0.60 p<0.0001	R^2^ = 0.37* CI95%–0.76–0.40 p<0.0001	R^2^ = 0.27* CI95%–0.70–0.29 p<0.0001	R^2^ = 0.23* CI95%–0.69–0.20 p = 0.001	R^2^ = 0.13* CI95%–0.66–0.6 p = 0.04	R^2^ = 0.09* CI95%–0.59–0.0 p = 0.04
**OSDI/Schirmer**	R^2^ = 0.32* CI95%–0.74–0.35 p<0.0001	R^2^ = 0.29* CI95%–0.71–0.31 p<0.0001	R^2^ = 0.05 CI95%–0.49–0.03 p = 0.08	R^2^ 0.04 CI95%–0.49–0.08 p = 0.14	R^2^ = 0.04 CI95%–0.53–0.18 p = 0.29	R^2^ = 0.02 CI95%–0.48–0.18 p = 0.32
**OSDI/Osmolarity**	R^2^ = 0.20* CI95% 0.19–0.66 p = 0.001	R^2^ = 0.17* CI95%0.15–0.62 p = 0.002	R^2^ = 0.24 CI95%–0.09–0.44 p = 0.16	R^2^ = 0.10* CI95%0.03–0.58 p = 0.02	R^2^ = 0.01 CI95%–0.47–0.27 p = 0.5	R^2^ = 0.15* CI95%0.07–0.64 p = 0.01
**Fluorescein/Lissamine**	R^2^ = 0.73* CI95%0.77–0.92 p<0.0001	R^2^ = 0.64* CI95%0.67–0.88 p<0.0001	R^2^ = 0.19* CI95% 0.18–0.64 p = 0.001	R^2^ = 0.30* CI95%0.29–0.73 p<0.0001	R^2^ = 0.17* CI95%0.06–0.69 p = 0.02	R^2^ = 0.59* CI95%0.59–0.87 p<0.001
**Fluorescein/TBUT**	R^2^ = 0.57* CI95%–0.86–0.62 p<0.0001	R^2^ = 0.44* CI95% –0.80–0.47 p<0.0001	R^2^ = 0.06 CI95% –0.49–0.03 p = 0.07	R^2^ = 0.015 CI95%–0.52–0.06 p = 0.103	R^2^ = 0.01 CI95%–0.48–0.25 p = 0.49	R^2^ = 0.11* CI95%–0.6–0.01 p = 0.03
**Fluorescein/Schirmer**	R^2^ = 0.60* CI95%–0.87–0.64 p<0.0001	R^2^ = 0.27* CI95%–0.70–0.28 p<0.0001	R^2^ = 0.002 CI95%–0.33–0.22 p = 0.69	R^2^ = 0.0006 CI95%–0.3–0.3 p = 0.95	R^2^ = 0.24* CI95%–0.73–0.5 p = 0.005	R^2^ = 0.04 CI95%–0.50–0.15 p = 0.24
**Fluorescein/Osmolarity**	R^2 = ^0.60* CI95%–0.87–0.64 p<0.0001	R^2^ = 0.18* CI95% 0.17–0.63 p = 0.0012	R^2^ = 0.06 CI95%–0.01–0.5 p = 0.05	R^2^ = 0.16* CI95%0.12–0.63 p = 0.005	R^2^ = 0.01 IC95%–0.44–0.28 p = 0.62	R^2^ = 0.16* CI95%0.08–0.6 p = 0.01
**Lissamine/TBUT**	R^2^ = 0.54* CI95%–0.85–0.58 p<0.0001	R^2^ = 0.32* CI95%–0.73–0.34 p<0.0001	R^2^ = 0.30* CI95%–0.72–0.32 p<0.0001	R^2^ = 0.14* CI95%–0.62–0.08 p = 0.01	R^2^ = 0.05 CI95%–0.56–0.15 p = 0.21	R^2^ = 0.08 CI95%–0.56–0.03 p = 0.07
**Lissamine/Schirmer**	R^2^ = 0.57* CI95% –0.86–0.61 p<0.0001	R^2^ = 0.09* CI95% –0.65–0.20 p = 0.0006	R^2^ = 0.23* CI95%–0.67–0.23 p = 0.0003	R^2^ = 0.08 CI95% –0.5–0.01 p = 0.05	R^2^ = 0.01 CI95%–0.48–0.24 p = 0.46	R^2^ = 0.04 CI95%–0.53–0.11 p = 0.17
**Lissamine/Osmolarity**	R^2^ = 0.20* CI95%0.18–0.66 p = 0.014	R^2^ = 0.24* CI95% 0,24–0.67 p = 0.0002	R^2^ = 0.04 CI95%–0.06–0.47 p = 0.11	R^2^ = 0.05 CI95%–0.06–0.5 p = 0.10	R^2^ = 0.0004 CI95%–0.34–0.39 p = 0.89	R^2^ = 0.09 CI95%–0.02–0.5 p = 0.06
**TBUT/Schirmer**	R^2^ = 0.50* CI95%0.53–0.82 p<0.0001	R^2^ = 0.54* CI95%0.58–0.84 p<0.0001	R^2^ = 0.23* CI95%0.23–0.67 p = 0.0003	R^2^ = 0.25* CI95%–0.33–0.76 p<0.0001	R^2^ = 0.19* CI95%0.08–0.69 p = 0.01	R^2^ = 0.25* CI95%0.26–0.75 p = 0.0005
**TBUT/Osmolarity**	R^2^ = 0.32* CI95%–0.74–0.34 p<0.0001	R^2^ = 0.25* IC95%–0.68–0.25 p = 0.0001	R^2^ = 0.03 CI95%–0.45–0.09 p = 0.16	R^2^ = 0.18* CI95%–0.65–0.14 p = 0.004	R^2^ = 0.04 CI95%–0.55–0.15 p = 0.22	R^2^ = 0.25* CI95%–0.74–0.27 p = 0.0003
**Schirmer/Osmolarity**	R^2^ = 0.13* CI95%–0.59–0.08 p = 0.010	R^2^ = 0.16* CI95%–0.61–0.14 p = 0.002	R^2^ = 0.10* CI95%–0.51–0.05 p = 0.01	R^2^ = 0.01 CI95% –0.41–0.19 p = 0.43	R^2^ = 0.002 CI95%–0.31–0.41 p = 0.77	R^2^ = 0.25* CI95%–0.75–0.25 p = 0.0006

Spearman's correlation coefficient, *p<0.05.

**Figure 1:**
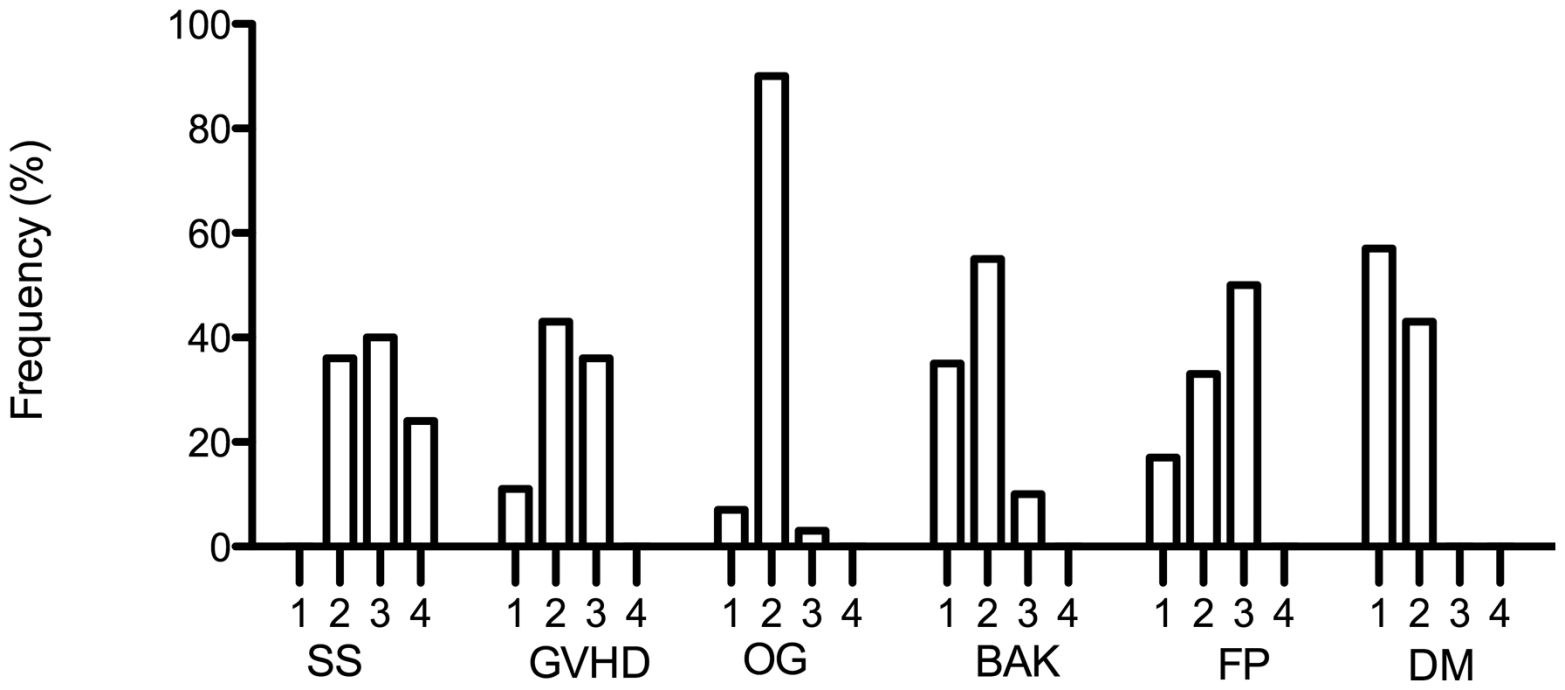
Severity grades among study groups. Frequency (%) of severity scores distribution among the study groups.

Most patients were classified as grades 2 and 3 in severity as it is shown in Figure 1. The SS patient subgroup had higher DED severity prevalence with a grade of 4 (24%). This same subgroup had among the four grades a more homogeneous DED score distribution. Results from all study parameters according to severity score showed a consistent association between severity scores and clinical and laboratory parameters.

Based on the arbitrary cut-off levels established here, the sensitivity, specificity, accuracy and positive and negative predictive values of the different diagnostic tests were evaluated. The most sensitive test was OSDI while the least accurate was lissamine green staining (Table 4). The test sensitivity among the different subgroups had a large variability (Table 5).

**Table 4:** Values of sensitivity, specificity, positive predictive value and accuracy for the tests of DED among the groups.

	OSDI	osmolarity	TBUT	Schirmer	Fluorescein	Lissamine
Sensitivity	84.2 (76.2–90.3)	40 (31.3–49.1)	72.3 (63.3–80.1)	56.6 (47.3–65.6)	38.1 (29.5–71.1)	25.6 (18.2–34.2)
Specificity	100 (82.4–100)	100 (83.2–100)	100 (83.2–100)	100 (83.2–100)	100 (83.2–100)	100 (83.2–100)
Accuracy	86.5 (79.5–91.8)	48.3 (39.9–56.7)	37.6 (24.8–52.)	62.7 (54.2–70.6)	46.6 (79.5–91.8)	35.9 (28.1–44.24
Positive Predictive Value	100 (96.2–100)	100 (92.8–100)	100 (95.8–100)	100 (94.8–100)	100 (92.6–100)	100 (89.1–100)
Negative Predictive Value	51.3 (34.4–68.1)	21.1 (13.3–30.6)	17.7 (11.2–26.0)	27.4 (17.6–70.6)	20.4 (12.9–29.7)	17.7 (11.2–26.0)

Data expressed in % and confidence interval of 95%.

**Table 5:** Values of sensitivity for the DED tests in the following conditions: SS, GVHD, Graves Orbitopathy, glaucoma treatment with BAK preserved eye drops, facial palsy and DM. Data expressed in % and confidence interval of 95%.

	Sjogren	GVHD	Graves	BAK	Facial palsy	DM
**OSDI**	96.1 (80.4–99.9)	81.5 (61.8–93.7)	91.3 (71.9–98.9)	76.5 (50.1–95.2)	83.3 (35.8–99.6)	69.2 (38.6–90.9)
**Osmolarity**	58.3 (36.6–77.9)	42.8 (24.5–100)	28.6 (13.2–48.7)	27.8 (9.7–53.4)	0 (0–34.8)	78.2 (49.2–100)
**TBUT**	96.1 (80.4–99.9)	81.5 (61.9–93.7)	67.8 (47.6–84.1)	64.3 (35.1–87.2)	66.7 (22.3–95.7)	46.1 (19.2–74.8)
**Schirmer test**	80.7 (60.6–93.4)	67.9 (47.6–100)	35.7 (18.6–55.9)	56.2 (29.9–80.2)	57.1 (18.4–90.1)	54.5 (23.4–83.2)
**Fluoresceine**	57.7 (36.9–76.6)	67.8 (47.6–84.1)	14.3 (4.0–32.7)	11.1 (1.37–34.7)	83.3 (35.8–99.6)	21.4 (4.67–50.1)
**Lissamine**	56 (34.8–75.6)	35.6 (18.5–55.8)	14.3 (4.0–32.7)	5.5 (0–27.3)	16.7 (0–64.12)	14.3 (1.8–42.8)

The best combination of tests to achieve the highest combined sensitivity (100%, C.I 95% 97.5–100), specificity (95%, C.I. 95% 75.1–99.9) and accuracy (99.3 C.I. 95% 96–99.9) for DED diagnosis was OSDI/TBUT/Schirmer test, based on the following probability calculation:
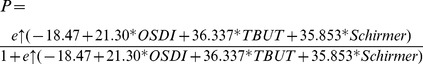



## Discussion

The present work revealed that there is very appreciable variation among diagnostic test results among different diseases and the best test combination to detect DED is OSDI/TBUT/Schirmer test. This result reinforces the importance of the most commonly used tests to detect DED in clinical practice, but also emphasizes their variability. By comparing distinctly related diseases to DED incidence, we found that those tests are poor predictors of this disease. This inadequacy makes it more apparent of the need to rely at this time on clinical interpretation of a combination of test results.

Meaningful diagnostic testing in DED patients across a broad range of different etiologies and presentations is still a challenge. [27], [28] Owing to the great variability in DED severity, it is unlikely that a single test result has adequate sensitivity to serve for DED given its multifactorial nature and numerous manifestations. It is important to consider, the overlap between normal and DED values, the lack of a gold standard test or even an ensemble of universally accepted tests and the lack of concordance between the signs and symptoms of this disease. Research on potential DED diagnostic tools and therapeutic agents has increased exponentially. [27], [29]–[31]

Even though there are a large number of symptoms as well as a wide range of methods and severity grades commonly linked to DED diagnosis, they can be also characteristic of other conditions besides DED.[29], [32] All subgroups reported higher OSDI scores compared to controls, although in chronically treated glaucoma patients and in diabetics they did not reach significance. OSDI scores correlated poorly with other tests in a broad analysis, but in SS patients the best agreement was found between: 1) OSDI and TBUT; 2) OSDI and fluorescein staining. Those findings suggest that when dryness reflects ocular surface changes, then neural pathways are better preserved than in patients with DM or BAK. In this situation, this association is consistent with patients' description of ocular discomfort.

TFBUT is a widely used test. It is minimally invasive, repeatable and more reliable than the Schirmer test. We found that the TFBUT had the greatest correlation with other tests in the different diseases. A possible reason for this agreement is that its score can vary depending on a larger number of factors, such as, exposed ocular surface area tear film volume and clearance among others and there is no widely accepted standard cut-off.

The ocular surface staining pattern is not necessarily altered in early stages of the disease. [33], [34] SS, GVHD and facial palsy patients had higher vital staining scores. Similar findings were obtained in a recent study comparing DED results in systemic conditions of Asian rheumatoid arthritic patients who had higher corneal staining scores than DM and smokers. [35]

Correlation coefficient analysis showed that the highest positive values were found between the two dye staining results. This was also the case with the group evaluation and between the SS and GVHD subgroups. As aqueous tears deficiency is characteristic of those diseases, this correlation agrees also with their correlation between lower Schirmer test and TFBUT values. The Schirmer test has been considered inaccurate, unrepeatable and not inclusive of the evaporative aspect of DED. [27]

Some studies have shown that tear film osmolarity is a feasible parameter for diagnosing and evaluating therapeutic response. [36] In addition, tear osmolarity measurements could have a parallel with DED severity. [25], [37] However, tear film osmolarity does not strongly correlate with other tests.[38] The present work shows, that SS, GVHD and DM without retinopathy patients also, presented with tear film hyperosmolarity, which reaffirms evidence, of its association with DED severity.

Of interest, the considerably higher tear film osmolarity mean values found in DM without retinopathy and those individuals under clinical treatment, where the DED severity was only 1 and 2, is in agreement with a report suggesting a possible influence of metabolic dysfunction on tear osmolarity. [39]

A critical appraisal of different DED causes in the present study reveals an overlap between test results due to the range of severity in the different categories as well as variations in the underlying pathophysiological mechanisms. SS and GVHD whose clinical DED findings allocated them as moderate to severe grades had higher test mean values and most significant correlations. Glaucoma patients included in this study were being chronically treated topically with one BAK preserved drug for at least one year. Other studies have shown that there is a high prevalence of ocular surface changes and symptoms in glaucoma patients whose severity correlates with the number of medications. [40], [41] DED frequency was high in DM without proliferative retinopathy patients, however in no case was DED severe or presenting with higher mean levels in other signs. This is in accordance with recent epidemiologic studies where proliferative retinopathy was associated with higher frequency and severity of DED. [42], [43]

DED is a serious and complex condition whose early recognition and treatment are crucial to avoiding losses in visual acuity and to improving quality of life. [44] On the basis of this study, we conclude that irrespective of the underlying causative DED condition, it remains quite difficult to interpret current DED diagnostic sets because of their frequent disagreement. Our results suggest that here is a lack of strong and consistent correlations among the test results. Herein, we could also observe that these values and severity grades ranged widely throughout the different disease subgroups. Nevertheless, each test could provide distinct information in a particular patient context, related diseases, risk factors and DED stage. It is reasonable that a specific test combination could provide better conclusions, regarding effective clinical management care. However, it remains very problematic to design meaningful clinical trials to evaluate the results of different DED treatment studies.

To determine the cut-off levels of tests for DED implies making a decision that is broad or restrictive. There is no widely accepted standard cut-off for its diagnosis.[6] Since our aims were to investigate the DED tests in pre-defined diseases with different mechanisms and to check whether any test has more accuracy in that specific group, we opted to define as a DED patient one who presented with changes in any of the tests in the panel, but to be more selective in terms of cut-off levels to avoid DED without sufficient cause.

In the future, a more comprehensive characterization of lacrimal gland dysfunction and/or ocular surface disease markers may provide valuable insight needed for a better understanding of underlying mechanisms needed for DED management.
